# Editorial: Hydrogen sulfide: Physiology, Pharmacology and Toxicology, Volume II

**DOI:** 10.3389/fphar.2022.943101

**Published:** 2022-06-29

**Authors:** Jin-Song Bian

**Affiliations:** Department of Pharmacology, School of Medicine, Southern University of Science and Technology, Shenzhen, China

**Keywords:** hydrogen sulfide, pharmacology, biology, cardiovascular, toxicology—metabolism, physiology

Hydrogen sulfide (H_2_S) is a reducing gas molecule in mammalian cells that can be endogenously synthesized by several enzymes, including cystathione β-synthase (CBS), cystathione γ-lyase (CSE), and 3-mercaptopyruvate sulfurtransferase (3-MST) (Sun et al., 2021). To date, a host of studies have revealed the great potential of H_2_S in preventing and treating a variety of human diseases by regulating numerous targets or signaling pathways (Rong et al., 2023). Despite our growing understanding of H_2_S-mediated biological and pharmacological effects on different systems, the precise underlying mechanisms responsible for the complicated effects of H_2_S have yet to be fully defined. As H_2_S is one of the important areas for the rapid development of gaseous signaling molecules, an overview of the results obtained in the past and future prospects may be highly desirable. To this end, we launched the Volume 2 of Hydrogen sulfide: Physiology, Pharmacology and Toxicology to highlight recent advances in our understanding of the physiological and pharmacological functions of H_2_S, including but not limited to biological function in the new compounds releasing H_2_S, polysulfide and SO_2_, new methods for detection of endogenous and exogenous H_2_S, advances in biosynthesis and metabolism of H_2_S, current understanding about protein sulfhydration, signaling mechanisms underlying the biological functions of H_2_S, prospect of sulfide-containing nature products and H_2_S synthesis inhibitors as therapeutic drugs. After the joint efforts of the journal, editors, reviewers, and contributors, a total of seven high-quality articles were received, including five original articles and 2 review articles. Although small in the kingdom of H_2_S, these seven articles have certainly contributed to advancing understanding of H_2_S biology and pharmacology. We have made a detailed summary and perspective for these seven articles as follows.

Hypothyroidism is characterized by the disruption of thyroid hormone synthesis and secretion by thyrocytes (Zhang et al., 2021). Sirtuin-1 (SIRT1) inactivation is involved in thyroid cell damage and thyroid hormone deficiency (Wei et al., 2022). H_2_S, as a gas signaling molecule, participates in many physiopathologic processes by upregulating SIRT1 (Yang et al., 2021). For this reason, Zhang’s group explored whether H_2_S promoted the synthesis and secretion of thyroid hormones by upregulating SIRT1 (Zhao et al.). It was found that serum levels of H_2_S were significantly downregulated in hypothyroid patients when compared to those of euthyroid participants (Zhao et al.). A H_2_S donor sodium hydrosulfide (NaHS) promoted thyroid function in hypothyroid rats and elevated the protein levels of TPO, NIS, Pendrin and MCT8 in human thyrocytes, an effect that was SIRT1-dependent, thus providing a new view of H_2_S for treating hypothyroidism (Zhao et al.). Through computer molecular docking technology, Geng’s group screened the natural small molecule compound norswertianolin (NW)-specific binding to CSE at Leu68 site (Niu et al.). Further studies disclosed that NW acting on the CSE/H_2_S system attenuated acute and long-term renal ischemia/reperfusion (I/R) injury, lowered blood pressure, ameliorated vascular remodeling and inflammation in rats (Niu et al.). Hence, NW may serve as a novel small molecular chemical compound CSE agonist, directly binding to CSE, heightening CSE generation–H_2_S activity, and then alleviating kidney I/R injury and hypertension (Niu et al.). In a study by Ni’s group, the authors identified that the arterial oxygen saturation (SaO_2_) was decreased in model of mouse with genetic deficiency of CSE, along with mild hypoxia occurred in the tissues of heart, lungs and kidneys in CSE^−/−^ mice (Huang et al.). H_2_S donor GYY4137 treatment increased SaO_2_ and ameliorated hypoxia state in cardiac and renal tissues (Huang et al.). Besides, they found the alveolar wall thickening, diffuse interstitial edema and leukocyte infiltration in pulmonary tissues and the increased inflammation and oxidative stress in the lung tissues of CSE-deficient mice, which were all ameliorated by GYY4137 treatment (Huang et al.). This observation indicated that endogenous H_2_S is an important factor in maintaining normal SaO_2_ by preventing oxidative stress and inflammation in the lungs.

The use of cyclosporine A (CsA) in transplant recipients is limited due to its side effects of causing severe hypertension (Pandey et al., 2022). CsA is reported to increase the activity of the epithelial sodium channel (ENaC) in cultured distal nephron cells, a potential event involved in CsA-induced hypertension (Wu et al., 2019). With this in mind, Ma’s research group investigated whether ENaC mediates CsA-induced hypertension and H_2_S could prevent such a type of hypertension (Wang et al.). They found that the open probability of epithelial sodium channel (ENaC) in principal cells of split-open cortical collecting ducts was significantly increased in rats after treatment with cyclosporine A (CsA), coinciding with intracellular reactive oxygen species (ROS) overproduction, such elevations were completely reversed by lovastatin (an inhibitor of cholesterol synthesis) or NaHS (a donor of H_2_S) (Wang et al.). Thereby, NaHS ameliorates CsA-induced hypertension by inhibition of oxidative stress (Wang et al.). In the cardiovascular system, endogenous H_2_S can lead to both vasodilatation and vasoconstriction (Mitidieri et al., 2021). Several mechanisms mediate vasodilatation induced by addition of exogenous H_2_S salts, including lowering of smooth muscle cells calcium by activation of K channels, enhancement of nitric oxide (NO) signaling, and changes in intracellular pH by inhibition of an acid-sensitive Cl2/HCO3-exchanger, to name a few (Meng et al., 2015). Abramavicius et al. tested the mechanisms involved in relaxation of small arteries induced by GYY4137. In pre-contracted small mesenteric arteries, GYY4137 induced concentration-dependent relaxations, which were inhibited by l-cysteine, blockers of large-conductance calcium-activated (BKCa) and voltage-gated type 7 (KV7) potassium channels (Abramavicius et al.). The perspective is that the rate of release of sulfides plays an important role for the effects of H_2_S salt vs. donors in small arteries, and hence for a beneficial effect of GYY4137 for treatment of cardiovascular disease.

My laboratory contributed a review article regarding the roles and molecular mechanism of H_2_S in cardiomyopathy and myocardial I/R injury under diabetes (Sun et al.). In this review article, we summarized the current findings on H_2_S biology and pharmacology, especially focusing on the novel mechanisms of H_2_S-based protection against diabetic cardiomyopathy and diabetes-aggravated cardiac I/R injury (Sun et al.). Li’s group reviewed the role of H_2_S in bone metabolism, and they summarized the current information about H_2_S donors related to bone metabolism diseases, such as osteoporosis and osteoarthritis, and discussed some mechanisms and biological applications (Hao et al.). Thus, H_2_S may be a novel regulator in bone metabolism (Hao et al.). Overall, these seven articles collected in this Research Topic highlighted the therapeutic potential of H_2_S in hypothyroidism, renal injury, vasodilation dysfunction, hypertensive vascular remodeling, lung inflammation, CsA-induced hypertension, diabetic cardiomyopathy, and bone disorders. H_2_S is considered an essential signaling molecule in the cardiovascular and nervous systems and a variety of pathophysiological changes. Despite the huge clinical value of H_2_S, we should be cautious while we are excited, especially considering the tissue specificity of H_2_S-producing enzymes, inconsistent and conflicting results of H_2_S, lower bioavailability, side effects, and even toxicity of H_2_S donors, lack of clinical practice on H_2_S. There is no doubt that the development of effective and long-lasting H_2_S donors is crucial for understanding the biological and pharmaceutical functions of H_2_S in various systems. We look forward to generating more interesting research in this area, and rapid clinical translation of H_2_S.
